# Decentralized automatic generation control of interconnected power systems incorporating asynchronous tie-lines

**DOI:** 10.1186/2193-1801-3-744

**Published:** 2014-12-16

**Authors:** Ibraheem, Naimul Hasan, Arkan Ahmed Hussein

**Affiliations:** Department of Electrical Engineering, Faculty of Engineering and Technology, Jamia Millia Islamia, New Delhi, India; Department of Electrical Engineering, Engineering College, Tikrit University, Tikrit, Iraq

**Keywords:** Automatic generation control, Genetic algorithm, Particle swarm optimization, Ziegler and Nichols method asynchronous tie-line

## Abstract

This Paper presents the design of decentralized automatic generation controller for an interconnected power system using PID, Genetic Algorithm (GA) and Particle Swarm Optimization (PSO). The designed controllers are tested on identical two-area interconnected power systems consisting of thermal power plants. The area interconnections between two areas are considered as (i) AC tie-line only (ii) Asynchronous tie-line. The dynamic response analysis is carried out for 1% load perturbation. The performance of the intelligent controllers based on GA and PSO has been compared with the conventional PID controller. The investigations of the system dynamic responses reveal that PSO has the better dynamic response result as compared with PID and GA controller for both type of area interconnection.

**Electronic supplementary material:**

The online version of this article (doi:10.1186/2193-1801-3-744) contains supplementary material, which is available to authorized users.

## 1. Introduction

Modern Power system consists of large number of generating units interconnected by transmission lines. The interconnection of the power systems enhance the stability and become a viable tool to provide the almost uninterruptible power to load canters from generating stations. The two power system areas may be connected through synchronous/asynchronous tie-lines. To provide a good quality of power, the operation of power system must be maintained at the nominal frequency and voltage profile. And it is achieved by controlling of real and reactive powers. A modern power system is divided into a number of control areas and each area is responsible for its own load and power interchanges. If the input-output power balance is not maintained, a change in frequency will occur which it highly undesirable. In modern interconnected power system, automatic generation control (AGC) is used to maintain the system frequencies and tie-line power flows at the specified nominal values.

The automatic generations control of interconnected power systems has become more significant as size and complexity of the system is going on increasing to meet out power demand. A large number of control techniques have been proposed by the researchers for the design of AGC regulators. In the early era, the AGC strategies were proposed base on centralized control strategy (Quazza^1966^; Elgerd & Fosha^1970^; Aldeen & Trinh^1994^; Fosha & Elgerd^1970^). The limitation of AGC centralized control strategy is that it requires the exchange of information from control areas spread over distantly connected geographical areas along with their increased computational and storage complexities**.** The decentralized automatic generation control strategies deals the limitations of centralized power system very effectively (Kawabata and Kido^1982^; Park & Lee^1984^; Calovic^1984^; Aldeen and Marsh^1990^,^1991^; Aldeen^1991^; Yang et al.^1998^,^2002^). The researchers (Kumar et al.^1985^) proposed the systematic distributed control design methods and achieved almost identical results as obtained with the centralized strategies. The design of decentralized load frequency controllers based on structured singular values and multiple control-structure constraints are discussed in (Kumar et al.^1987^; Shayeghi et al.^2007^). The decentralized AGC regulator design based on the structured singular value is designed for local area robust analysis, and an eigen value method is derived for tie-line robustness analysis (Tan & Zhou^2012^). (Tan^2011^) proposed a method to analyze the stability of multi-area power system by accounting the inherent structure of the multi-area power system. (Sudha and Vijaya Santhi^2011^) proposed a Type 2 Fuzzy controller for decentralized two area interconnected power system with consideration of generation rate constraint.

## 2. Power system models

In this paper two power system models are considered for design of decentralized AGC regulators using PID, GA and PSO. The area interconnection in one power system model is only AC tie line and in the second model Parallel AC/DC link is considered

## 3. State space model

The linear time-invariant state space representation of interconnected power system is given by the following equations:

1 $$\dot{X}=\mathrm{AX}+BU+\Gamma D$$

2 Y=Cx

Where A, B, Γ are system, control and disturbance matrices and x, u and d are system control and disturbance vectors.

*Power system model–I:*

$$\begin{array}{l} \left[X_{1}]{}^{T}=[\Delta f_{1}\Delta P_{t1}\Delta P_{g1}\Delta f_{2}\Delta P_{t2}\Delta P_{g2}\Delta {P_{\mathrm{tie}}}_{12}\mathrm{AC}E_{1}\mathrm{AC}E_{2}\right]\\ \;\left[U_{1}\right]=\left[u1\;u2\left[,\;\left[D_{1}\right]=\right]\Delta P_{d1}\;\Delta P_{d2}\right] \end{array}$$

*Power system model–II*

$$\begin{array}{l} {\left[X_{2}\right]}^{T}=\left[\Delta f_{1}\;\Delta P_{t1}\Delta P_{g1}\;\Delta f_{2}\;\Delta P_{t2}\Delta P_{g2}\Delta P_{\mathrm{tie}12}\;\mathrm{AC}E_{1}\;\mathrm{AC}E_{2}\;\Delta P_{\mathrm{dc}}\right]\\ \left[U_{2}\right]=\left[U_{1}\right],\;\left[D_{2}\right]=\left[D_{1}\right] \end{array}$$

*State equations:*

**Figure 1:**
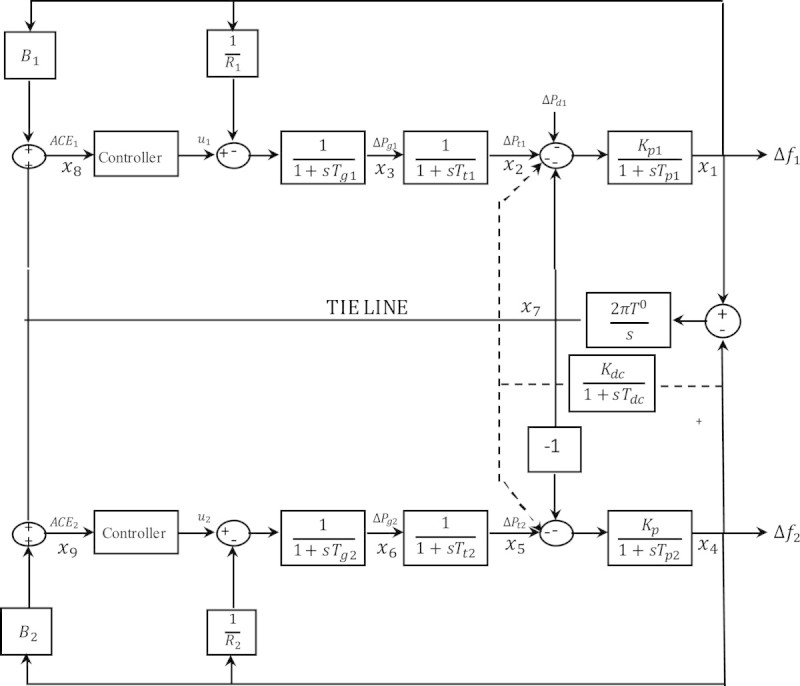
**Transfer function model of power system.**

From the transfer function block diagram shown in Figure 1, the following equations are obtained:

3 $${\dot{x}}_{1}=-\frac{1}{T_{p1}}x_{1}+\frac{K_{p1}}{T_{p1}}{\dot{x}}_{2}-\frac{K_{p1}}{T_{p1}}x_{7}-\frac{K_{p1}}{T_{p1}}\Delta P_{d1}$$

4 $${\dot{x}}_{2}=-\frac{1}{T_{t1}}{\dot{x}}_{2}+\frac{1}{T_{t1}}x_{3}$$

5 $${\dot{x}}_{3}=-\frac{1}{R_{1}T_{g1}}{\dot{x}}_{1}-\frac{1}{T_{g1}}x_{3}+\frac{1}{T_{g1}}u_{1}$$

6 $${\dot{x}}_{4}=-\frac{1}{T_{p2}}x_{4}+\frac{K_{p2}}{T_{p2}}x_{5}+\frac{K_{p2}}{T_{p2}}x_{1}-\frac{K_{p2}}{T_{p2}}\Delta P_{d2}$$

7 $${\dot{x}}_{5}=-\frac{1}{T_{t2}}x_{5}+\frac{1}{T_{t2}}x_{6}$$

8 $${\dot{x}}_{6}=-\frac{1}{R_{2}T_{g2}}x_{4}-\frac{1}{T_{g2}}x_{6}+\frac{1}{T_{g2}}u_{2}$$

9 $${\dot{x}}_{7}=2\pi T^{0}x_{1}-2\pi T^{0}x_{3}$$

10 $${\dot{x}}_{8}=B_{1}x_{1}+x_{7}$$

11 $${\dot{x}}_{9}=B_{2}x_{4}-x_{7}$$

From the above equations, System, control and disturbance matrices can be obtained as given below:

State matrix ‘A’, Control matrix ‘B’, and disturbance matrix Γ for power system model-I are as follows below. The same matrices can be obtained for the power system model-II.

$$\begin{array}{l} A=\left[\begin{array}{ccccccccc} -1/Tp1 & Kp1/Tp1 & 0 & 0 & 0 & 0 & -Kp1/Tp1 & 0 & 0\\ 0 & -1/Tt1 & 1/Tt1 & 0 & 0 & 0 & 0 & 0 & 0\\ -1/R1\mathrm{Tg}1 & 0 & -1/\mathrm{Tg}1 & 0 & 0 & 0 & 0 & 0 & 0\\ 0 & 0 & 0 & -1/Tp2 & Kp2/Tp2 & 0 & Kp2/Tp2 & 0 & 0\\ 0 & 0 & 0 & 0 & -1/Tt2 & 1/Tt2 & 0 & 0 & 0\\ 0 & 0 & 0 & -1/R2\mathrm{Tg}2 & 0 & 1/\mathrm{Tg}2 & 0 & 0 & 0\\ 2\pi T^{0} & 0 & 0 & -2\pi T^{0} & 0 & 0 & 0 & 0 & 0\\ B1 & 0 & 0 & 0 & 0 & 0 & 0 & 0 & 0\\ 0 & 0 & 0 & B2 & 0 & 0 & 0 & 0 & 0 \end{array}\right]\\ B=\left[\begin{array}{cc} 0 & 0\\ 0 & 0\\ 1/Tg_{1} & 0\\ 0 & 0\\ 0 & 0\\ 0 & 1/Tg_{2}\\ 0 & 0\\ 0 & 0\\ 0 & 0 \end{array}\right]\;\Gamma =\left[\begin{array}{cc} -Kp_{1}/Tp_{1} & 0\\ 0 & 0\\ 0 & 0\\ 0 & -Kp_{2}/Tp_{2}\\ 0 & 0\\ 0 & 0\\ 0 & 0\\ 0 & 0\\ 0 & 0 \end{array}\right] \end{array}$$

The area control error for area-1 is defined as:

12 $$\mathrm{AC}E_{1}=\Delta P_{\mathrm{tie}1}+B_{1}\Delta f_{1}$$

and the feedback control for Area-1 takes the form

13 $$u_{1}=-K_{1}\left(s\right)\mathrm{AC}E_{1}$$

where K_1_(s) is the local LFC controller for area-1.

According to (Tan^2009^,^2010^), a decentralized controller can be designed assuming that there are no tie-line power flows, In this case the local feedback control will be

14 $$u_{1}=-K_{1}\left(s\right)B_{1}\Delta f_{1}$$

## 4. A control scheme for an interconnected power system

### 4.1 Tuning of AGC parameter

The AGC regulator has the objective to minimize area control error (Xue et al.^2007^). The AGC regulators having single output as a control signal based on PID is given below;

15 $$u\left(t\right)=K_{P}\left[e\left(t\right)+\frac{1}{T_{i}}\int_{0}^{t}e\left(\tau \right)d\tau +T_{d}\frac{de\left(t\right)}{dt}\right]$$

where *u(t)* is the control input for governor , e(t) the error , The tuning process of PID controller gain is done by Ziegler and Nichols (ZN) method (Asttrom & Hagglund^1995^). The proportional, integral and derivative gains are calculated for the critical ultimate gain, Ku and oscillation of ultimate time period, Tu. These gains are shown below in Table 1.

**Table 1:** **Gains of PID controller**

Controllers	Proportional gain	Integral gain	Derivative gain
P	0.5 Ku		
PI	0.4 Ku	0.8 Tu	
PID	0.6 Ku	0.5 Tu	0.12 Tu

### 4.2 Genetic algorithm

The genetic algorithm is a nature inspired optimization technique (Goldberg^1989^). There are some sequential steps to be followed in developing the GA for automatic generation control. The Chromosomes Structure is built up with the initial set of random population in the form chromosomes which consists of genes as binary bits. These binary bits are then decoded to give proper string for optimization. The new population are regenerated which is to be converged at global optimum by the specified selection, crossover and mutation operators. Elitism is applied to save and use previously found best partner in subsequent fittest generation of population.

The processes stop as soon as convergence criterion is satisfied.

**Figure 2:**
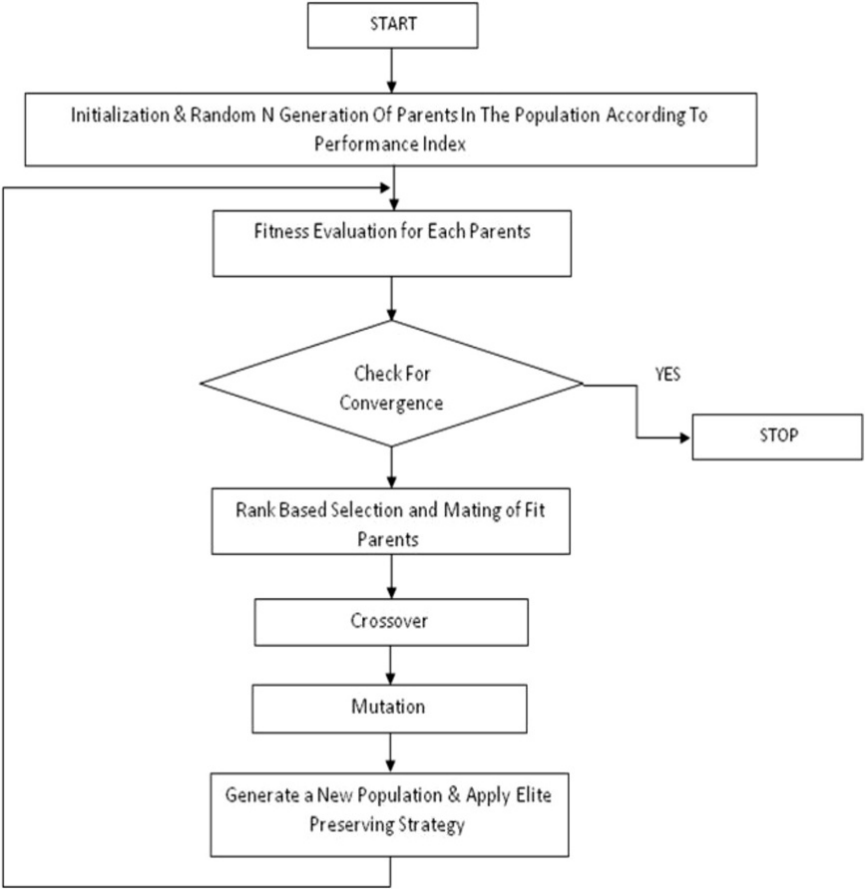
**Flowchart of GA based optimization technique.**

The flow chart of the GA algorithm used in this work is shown in Figure 2.

### 4.3 Particle Swarm Optimization (PSO)

Particle swarm optimization (PSO) is a population-based stochastic optimization technique which is based on the social behavior of bird flocking, fish schooling and swarming theory (Kennedy & Eberhart^1995^; Eberhart & Kennedy^1995^). In the PSO method, a swarm consists of a set of individuals named as particles are specified by their position and velocity vectors (x_i_(t), v_i_(t)) at each time. In an n-dimensional solution space, each particle is treated as an n-dimensional space vector and the position of the i^th^ particle is presented by x_i_ = [x_i_ (1), xi(2), …, xi(n)]; then it flies to a new position by the velocity represented by v_i_ = [v_i_(1), v_i_(2), …, v_i_(n)]. The best position for i^th^ particle represented by p_best,i_ = [p_best,i_(1), p_best,i_(2), …, p_best,i_(n)] is determined according to the best value for the specified objective function and this global best position is represented as g_best_ = (g_best,_1, g_best,_2, …, g_best,_n). For the next iteration, the position x_ik_ and velocity v_ik_ corresponding to the k^th^ dimension of i^th^ particle are updated using the following equations:

16 $$\begin{array}{ll} \;v_{ik}\left(t+1\right) & =w.v_{ik}+c_{1}.\mathrm{ran}d_{1,ik}\left(p_{\mathrm{best},ik}\left(t\right)\right)\\ \;+c_{2}.\mathrm{ran}d_{2,ik}\left(g_{\mathrm{best},k}\left(t\right)-x_{ik}\left(t\right)\right) \end{array}$$

17 $$x_{i,k}\left(t+1\right)=x_{ik}\left(t\right)+v_{ik}\left(t+1\right)$$

where i = 1, 2, …, n is the index of particles, w is the inertia weight, rand_1,ik_ and rand_2,ik_ are random numbers in the interval [0 1], c_1_ and c_2_ are learning factors, and t represents the iterations.

**Figure 3:**
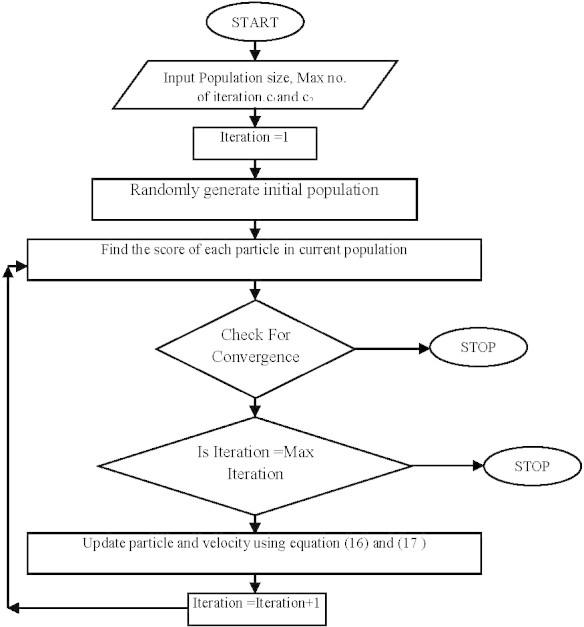
**Flowchart of PSO optimization technique.**

The flow chart of PSO as implemented for optimization is shown in Figure 3.

## 5. Simulation results and discussion

**Figure 4:**
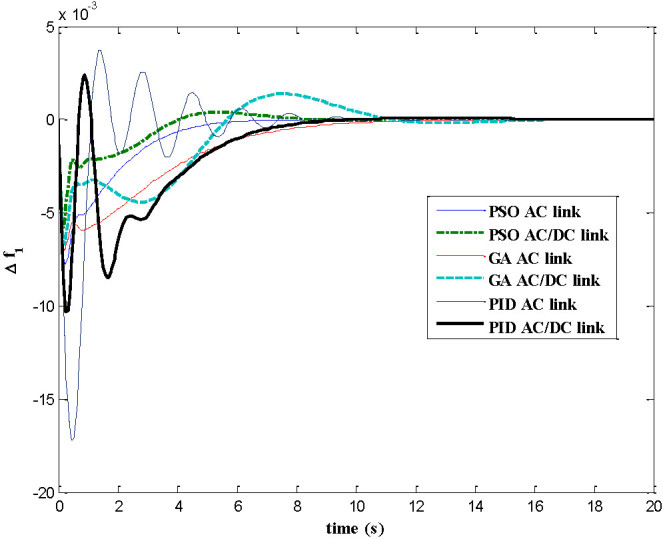
**Dynamic response for ∆f**_**1**_ **.**

The dynamic responses of various system states of interconnected decentralized power system are obtained for AGC regulators designed using PID, GA and PSO. The simulation work is carried out using MATLAB software with numerical data shown in appendix A. In this paper both AC tie-line and parallel AC/DC tie-line as area interconnection are considered for the investigations. The time responses are plotted for various system states with implementation of designed AGC regulators considering 1% load perturbation in area-1. The Figures 4 and5 show the dynamic responses of the frequency deviations in area-1 and area-2 respectively. The investigations of these plots inferred that with PSO controller, the oscillation, overshoot decreases as compared with GA and PID controller and also the settling time is faster in the case of time response with PSO with AC/DC tie-line compared to those offered by GA and PID. Figure 6 represents the tie-line power flow deviation between the two areas. The analysis reveals that the proposed controllers are capable to mitigate the deviations in tie-line power flows. The PSO controller has the superiority to the GA and PID in terms of over shoots and settling time. The Figures 7 and8 are plotted for the area control error for area 1&2 respectively, the Figure 7 shows that the PSO controller has the best over shoot and settling time. The Figure 8 shows that the overshoot and settling time with GA controller is comparable with PSO and PID.

**Figure 5:**
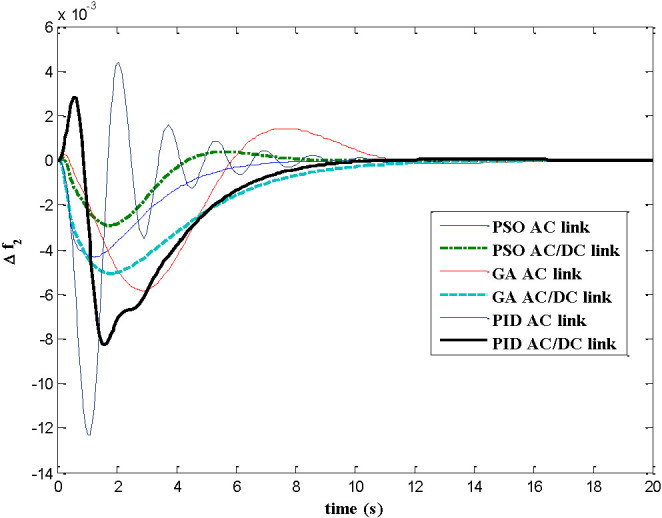
**Dynamic response for ∆f**_**2**_ **.**

**Figure 6:**
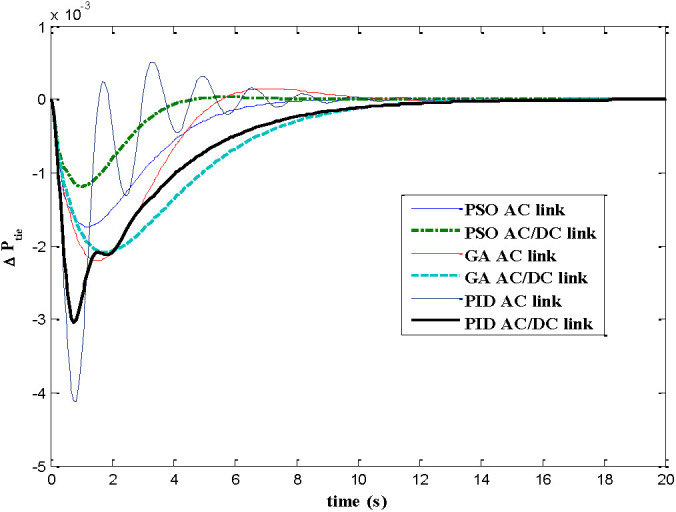
**Dynamic response for P**_**tie12**_ **.**

**Figure 7:**
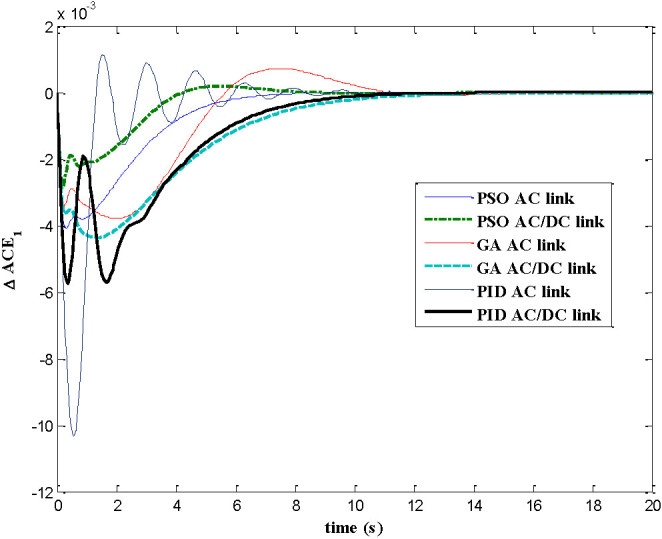
**Dynamic response for ACE**_**1**_ **.**

**Figure 8:**
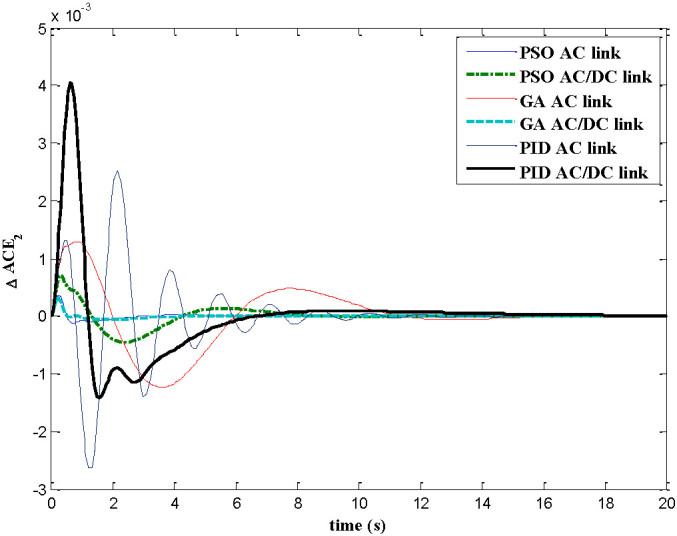
**Dynamic response for ACE**_**2**_ **.**

## 6. Conclusion

The AGC regulators are designed using PID, GA and PSO for two-area interconnected decentralized power system. The area interconnections are considered as AC tie-line and parallel AC/DC tie-lines. Investigations of results are presented that inferred the superiority PSO controller in comparison to PID and GA. The comparisons have been made between the power system model-I and power system model-II consisting of AC tie-line and parallel AC/DC tie-line. The positive effect of DC link in parallel to AC tie-line is also clearly visible in the time response plots of all states with the designed regulators.

## Nomenclature

*i* subscript referring to area *i* (*i* = 1,2)

Δ*f*_*i*_ frequency deviation of Area (Hz)

*ACE*_*i*_ area control error,

Δ*P*_*ti*_ incremental change in power generation,

ΔP_gi_ incremental change in governor valve position,

Δ*P*_*tie*_ tie-line power deviation,

*T*_*gi*_ governor time constant for the ith area subsystem (s),

*T*_*ti*_ turbine time constant for the ith area subsystem (s),

*T*_*pi*_ plant model time constant for the ith area subsystem (s),

*T*_*ij*_ synchronizing coefficient between the ith and jth area subsystem (p.u. MW),

*K*_*pi*_ plant gain for the ith area subsystem,

*R*_*i*_ speed regulation due to governor action for the ith area subsystem,

χ_*i*_ (*t*) states of the i^th^ area subsystem,

*u*_*i*_(*t*) control input for the ith area subsystem.

ZN Ziegler and Nichols control method

ACE Area Control Error

## Authors’ original submitted files for images

Below are the links to the authors’ original submitted files for images. Authors’ original file for figure 1 Authors’ original file for figure 2 Authors’ original file for figure 3 Authors’ original file for figure 4 Authors’ original file for figure 5 Authors’ original file for figure 6 Authors’ original file for figure 7 Authors’ original file for figure 8
