# Supplementary figures and images for: Decentralized automatic generation control of interconnected power systems incorporating asynchronous tie-lines
Source: Springerplus. 2014 Dec 16;3:744. doi: 10.1186/2193-1801-3-744 (PMC4320131; doi:10.1186/2193-1801-3-744)

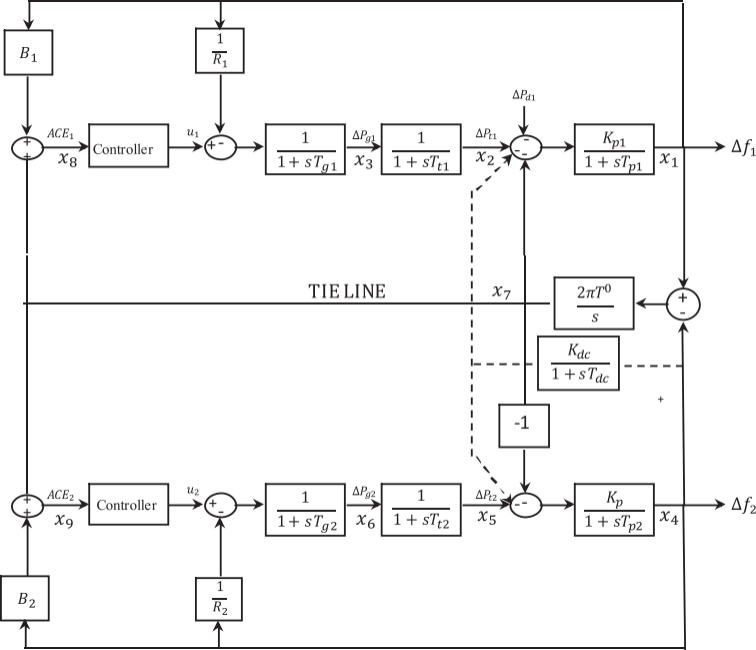

Supplement: Supplementary file 1 — Authors’ original file for figure 1 [file 40064_2014_1520_MOESM1_ESM.pdf]

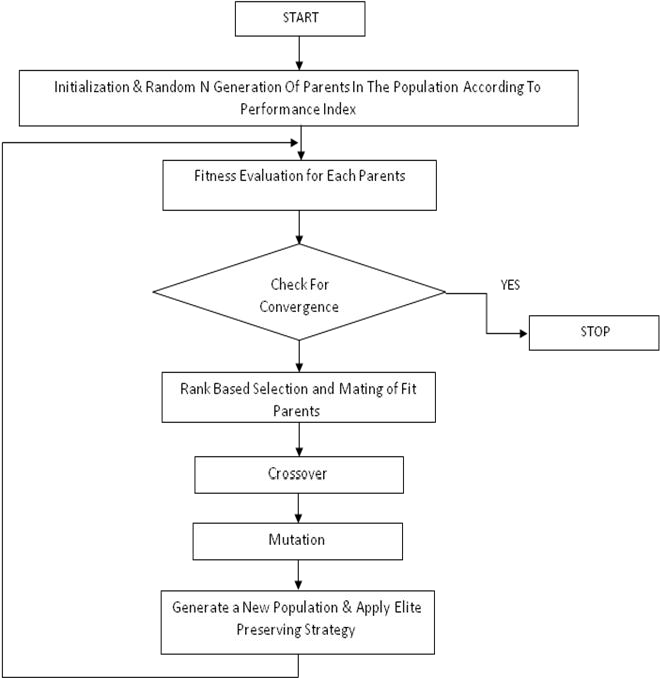

Supplement: Supplementary file 2 — Authors’ original file for figure 2 [file 40064_2014_1520_MOESM2_ESM.tiff]

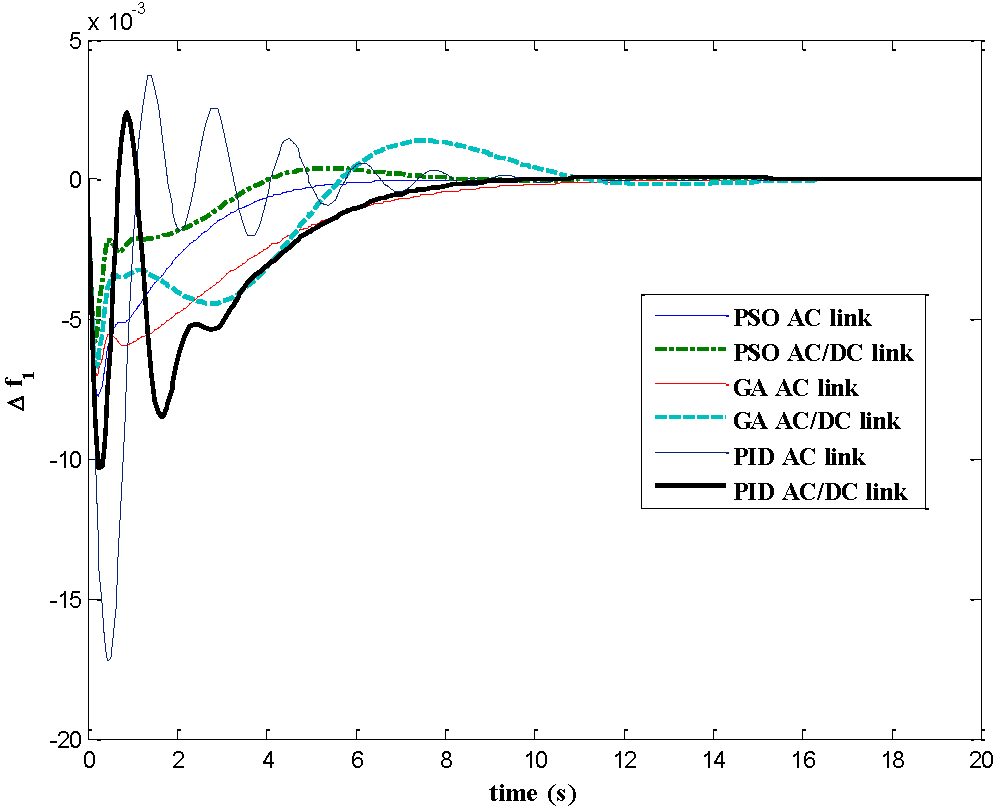

Supplement: Supplementary file 4 — Authors’ original file for figure 4 [file 40064_2014_1520_MOESM4_ESM.tiff]

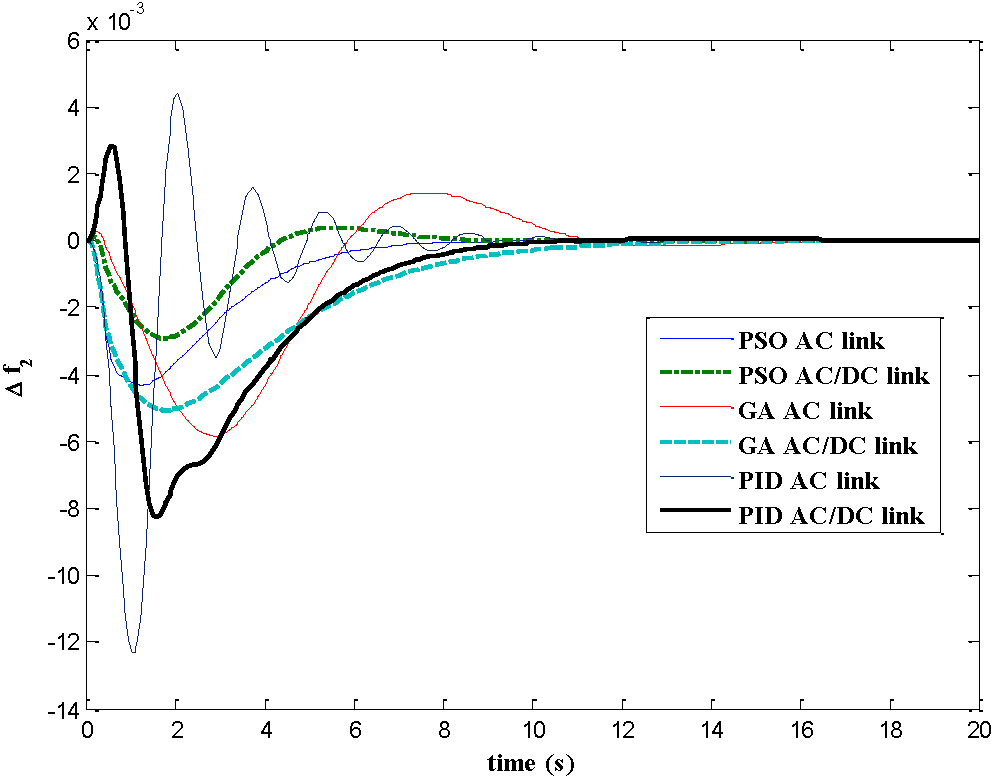

Supplement: Supplementary file 5 — Authors’ original file for figure 5 [file 40064_2014_1520_MOESM5_ESM.tiff]

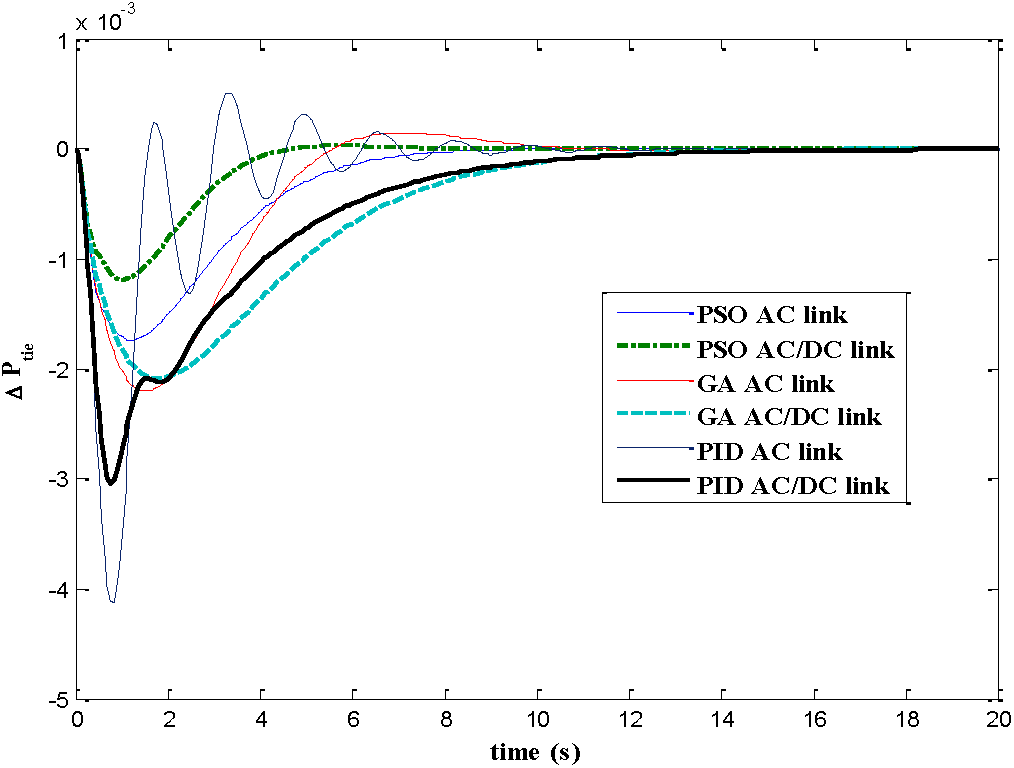

Supplement: Supplementary file 6 — Authors’ original file for figure 6 [file 40064_2014_1520_MOESM6_ESM.tiff]

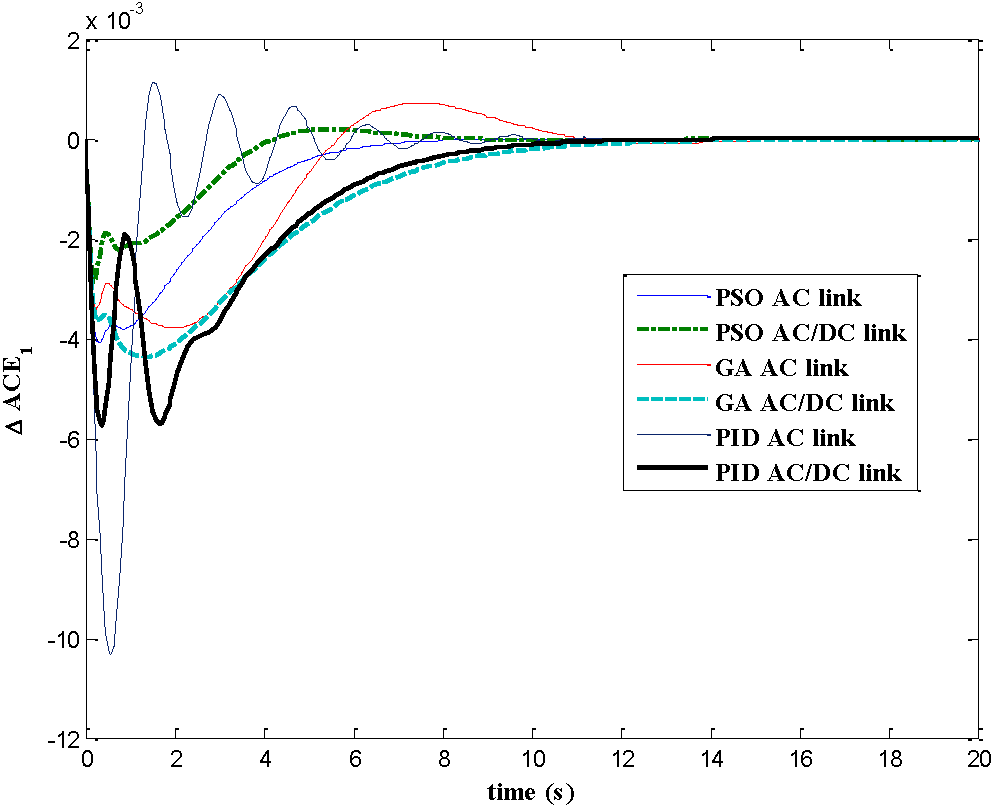

Supplement: Supplementary file 7 — Authors’ original file for figure 7 [file 40064_2014_1520_MOESM7_ESM.tiff]

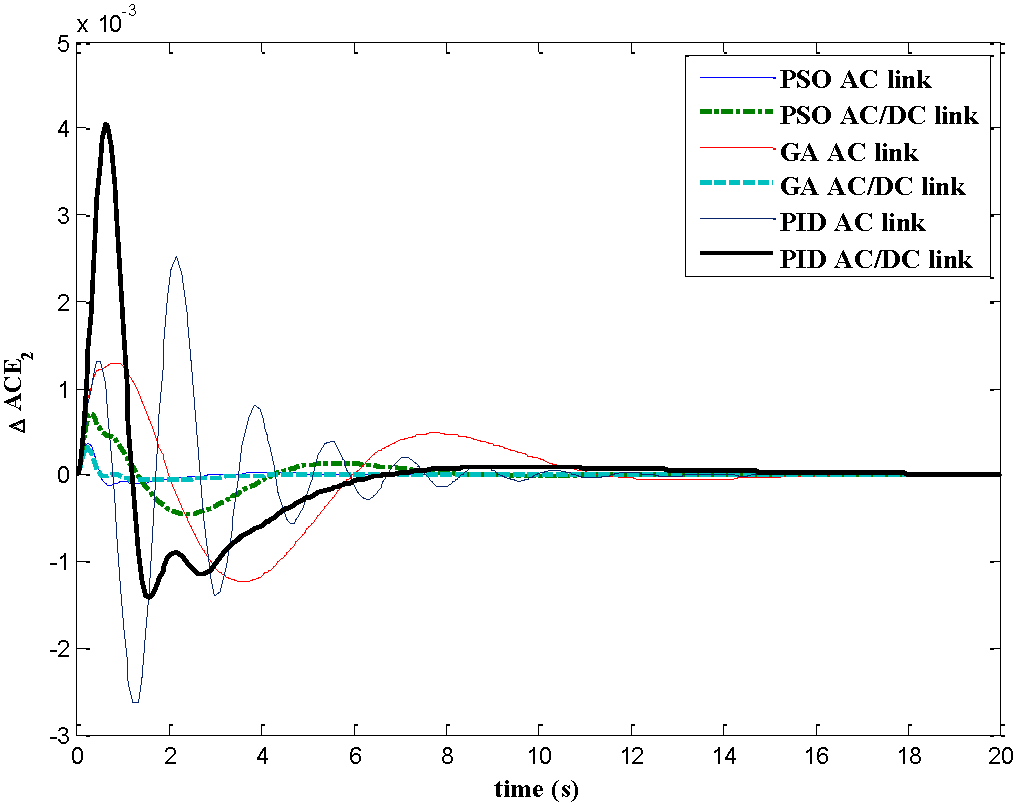

Supplement: Supplementary file 8 — Authors’ original file for figure 8 [file 40064_2014_1520_MOESM8_ESM.tiff]
